# Identification of IV fluid contamination in complete blood counts and subsequent unnecessary red blood cell transfusions using artificial intelligence

**DOI:** 10.1111/trf.70072

**Published:** 2026-01-08

**Authors:** Carly Maucione, Nathan McLamb, Mark A. Zaydman, Lauren N. Pearson, Ryan A. Metcalf, Nicholas C. Spies

**Affiliations:** ^1^ Department of Pathology and Immunology Washington University School of Medicine St. Louis Missouri USA; ^2^ Department of Pathology University of Utah Health Salt Lake City Utah USA; ^3^ ARUP Laboratories Salt Lake City Utah USA

## Abstract

**Background:**

Specimens contaminated with intravenous (IV) fluids can lead to considerable measurement errors in complete blood counts (CBCs), posing challenges for laboratory operations and clinical decision‐making. There is no gold‐standard method for retrospectively identifying these events, making it difficult to target quality improvement initiatives or optimize laboratory detection protocols.

**Study Design and Methods:**

This study aimed to develop and validate machine learning (ML) models to retrospectively identify IV fluid contamination in CBC results at scale across two institutions. The models were trained on simulated contamination in CBCs using prior, current, and post hemoglobin concentrations, platelet counts, and white blood cell counts, then validated against expert‐reviewed datasets. Real‐world applicability was assessed using 1 year's worth of CBC results from each institution.

**Results:**

The models effectively discriminated contaminated from non‐contaminated results on real‐world datasets, with areas under the receiver operating characteristic curve of 0.972 and 0.957, and areas under the precision‐recall curve of 0.723 and 0.619. In 1 year of CBC data, ~2% of inpatient CBC trios were predicted as contaminated across both institutions. 6%–9% of inpatient transfusions for which a CBC trio was available were deemed potentially unnecessary using a rule set validated by expert chart review.

**Discussion:**

The findings support the feasibility of using ML to identify IV fluid contamination in CBC results efficiently and effectively. Further work, including prospective real‐world evaluations, targeted quality improvement initiatives, and development of real‐time detection models, is necessary before realizing the potential benefits to patient safety, laboratory operations, and patient blood management.

## 1 INTRODUCTION

Specimens collected directly from, or downstream of, an intravenous (IV) catheter can demonstrate substantial measurement error. Dubbed “IV fluid contamination”, these events cause operational burdens in the laboratory and disruptions in clinical workflows when detected by routine quality safeguards. Worse, however, is the risk of preventable patient harm when errors go undetected, prompting inappropriate clinical decisions motivated by inaccurate results. Current protocols for detecting these errors in real time are woefully inadequate,^1^, ^2^, ^3^, ^4^, ^5^ and there is no gold‐standard method for identifying these events retrospectively, making objective characterization of the problem difficult.

IV fluid contamination often causes an “anomaly‐with‐resolution” pattern across all measured analytes. These patterns depend upon the contaminating fluid's composition, as well as its relative abundance in the specimen (the “mixture ratio”). As such, for a contaminated CBC, all count and concentration results should initially decrease, then rebound toward the patient's baseline when a subsequent specimen is properly collected. These predictable patterns are ideal for machine learning (ML) applications. As such, ML models have demonstrated success in other preanalytical errors on CBC results,^6^, ^7^ and on IV fluid contamination in chemistry results,^8^, ^9^, ^10^, ^11^, ^12^, ^13^, ^14^ but whose potential has yet to be evaluated for contaminated CBCs.

Red blood cell (RBC) transfusion is a common intervention for symptomatic acute blood loss anemia, though it is not without risk. For patients, transfusion reactions occur in about 0.1%–0.2% of RBC transfusions, ranging from asymptomatic alloimmunization to life‐threatening anaphylaxis or hemolysis.^15^ For hospitals and laboratories, the logistic and operational infrastructure required to provide transfusion services is substantial. Additionally, each unit confers direct acquisition costs to the laboratory, with the median leukoreduced RBC unit priced around $210 USD.^16^ Evidence‐based clinical practice guidelines offer several clinical decision limits for the hemoglobin concentrations at which RBC transfusion is justified.^17^

Prior work has evaluated random samples of 1000 CBC results and 1000 transfusions across multiple institutions, using expert review to define contamination events and potentially unnecessary transfusions.^18^ In that study, IV fluid contamination was estimated to have occurred in ~4% of inpatient CBC trios, and in the specimen prior to ~12% of single‐unit RBC transfusions (for which a CBC trio was available). Per review of the electronic medical record, ~8% of these 1000 transfusions were deemed “potentially unnecessary”, in that they were likely motivated by dilutional effects of contamination, and not by other relevant clinical factors.

These findings underscore the need for more comprehensive evaluation of this potential problem. However, this retrospective chart review approach is labor‐intensive and prone to intra‐ and inter‐reviewer variation. A machine learning approach could provide a scalable and generalizable mechanism for efficiently evaluating this problem.

This work presents the development and validation of a proof‐of‐concept machine learning solution for the retrospective identification of IV fluid contamination in CBC results. We evaluated model performance using labels curated by extensive manual chart review, then interrogated these models using explainability techniques. Finally, we applied the validated models to 1 year of real‐world data, estimating the prevalence of these events, their potential impact on patient blood management, and the opportunities for more targeted quality improvement interventions.

## 2 MATERIALS AND METHODS

### 2.1 Data collection

This study was approved by Institutional Review Boards at each institution (IRB 00185418 and 202410120). For the University of Utah (*“Utah”*), hemoglobin concentration (*Hgb*), platelet counts (*Plt*), and white blood cell counts (*WBC*) were extracted from the enterprise data warehouse at ARUP Laboratories. For Washington University School of Medicine (*“WashU”*), the same features were extracted from the laboratory information system of Barnes‐Jewish Hospital and St. Louis Children's Hospital. Given the collinearity between hemoglobin concentration and hematocrit percentage, only hemoglobin was included in model development.

Deltas were calculated using the most recent result in prior and subsequent draws for that analyte within 48 h. If none existed, that row was removed. These complete sets of current CBC results, their changes from prior, and their changes to post will be referred to as a *“CBC trio”*. While no explicit patient‐based inclusion/exclusion criteria were applied, this trio‐based filtering likely excludes the majority of outpatient specimens. Results reported with a “>” or “<” were replaced with their corresponding numerical values (e.g., 1500 k/cumm, rather than >1500). Any result that could not be coerced into a numerical value was dropped (e.g., “See Comment”).

RBC transfusions were extracted via SlicerDicer (Epic systems, Verona, WI), using the blood product administration modules. We estimate that around 90% of all transfused units were reliably captured with this approach, but will not capture units given as emergency release (massive transfusion protocols, whole blood, and custom order sizes) or those with scanning errors.

### 2.2 Algorithm development

**FIGURE 1:**
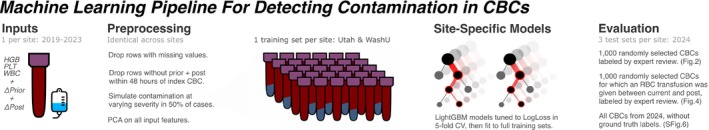
Schematic overview of the machine learning approach. CBC, complete blood count; CV, cross‐validation; HGB, hemoglobin; PCA, principal component analysis; PLT, platelet count; WBC, white blood cell count.

A schematic overview of the methods is provided in Figure 1. Machine learning (ML) pipelines were developed independently for each institution. CBC results from 2021 to 2023 were partitioned into training sets (278,447 trios at Utah, 945,736 at WashU), while results from 2024 were set aside as independent test sets (110,299 and 322,025). Contamination was simulated as a linear dilution of all analytes in a random selection of 50% of CBC trios, with all non‐simulated trios labeled negative for contamination. This simulation was validated using serial dilutions of real‐world patient specimens (Supporting Information Figure 1). Contamination severities were drawn from a distribution of mixture ratios that mimicked real‐world contamination events^5^ (Supporting Information Figure 2).

The ML pipeline was developed using R 4.3.2 and the *{tidymodels}* ecosystem.^19^, ^20^, ^21^, ^22^, ^23^, ^24^ Input features included the current Hgb, Plt, and WBC, as well as their relative changes when compared to their most recent prior and subsequent results for each patient. Given that the true error‐generating process (dilution by contaminating fluid) is linear and proportional across all analytes, we hypothesized that principal component analysis (PCA) would effectively capture the resulting anomaly‐with‐resolution pattern from diluted specimens. However, residual biological and analytical variation will remain after the variance in this dilutional effect is captured by principal components. As such, we opted to also include the raw feature values in addition to the principal components, with short tree depths and high splitting fractions to add robustness to overfitting from the simulated training set. To test these hypotheses, three feature sets were evaluated: features alone, principal components alone, and a combined approach. Final models were fit to the full training sets before being applied to the independent test sets.

### 2.3 Performance assessment

Two previously described expert‐labeled validation sets from each institution were used to assess model performance.^18^ The first included 1000 randomly selected CBC trios, without exclusions, to provide an unbiased performance assessment. The second included 1000 trios for which a RBC transfusion was administered between the current and post CBC was collected to evaluate if the model's inferences were robust to transfusion‐induced hemoglobin increases.

All performance assessments and summary figures were generated from each model's predictions on their institution's respective validation sets. A binary classification threshold was applied such that a positive predictive value of >0.80 would be achieved, which corresponded to a classification threshold of 0.75 in the model output. Performance metrics were calculated using the labels from manual chart review as the ground truth. Summary tables were generated using *{gtsummary}*.^23^ Global and local explanations of feature importance were estimated using Shapley additive explanation (SHAP) values.^25^ To facilitate replication and reproduction, anonymized data is available on FigShare at https://doi.org/10.6084/m9.figshare.30408268.v1 and analysis code is available on GitHub at https://github.com/nspies13/transfusions_from_contaminated_cbcs.

### 2.4 Evaluating CBC contamination prevalence across contexts

To evaluate contamination across various clinical and patient contexts, the validated models were applied retrospectively to one full year of CBC results from each institution. Contamination severity was evaluated by estimating the most likely mixture ratio of contaminating fluid relative to the physiologic patient specimen using a regression‐based approach.^13^

To provide orthogonal evidence supporting the predictions, CBC trios predicted as contaminated at a mixture ratio above 0.10 were matched to any corresponding basic metabolic panel (BMP) that was collected within 5 min of the current CBC. Mixture ratios were estimated in the corresponding BMPs using previously validated BMP‐specific models,^13^ then compared in a scatterplot.

### 2.5 Assessing impact on RBC transfusion decisions

Additional evaluation is necessary to assess whether a RBC transfusion that is given after a contaminated CBC is potentially unnecessary. To differentiate medically justified transfusions from those that were potentially unnecessary (motivated by the dilutional effect of contamination), the following criteria were developed using the validation sets of 1000 transfusions, then applied to the full year of transfusion administrations:

1. The current CBC was predicted to be contaminated by the models;
2. The post‐transfusion Hgb was >8 g/dL, to remove most cases for which empiric transfusion may be justified by AABB guidelines^17^;
3. The post‐transfusion hemoglobin was greater than both the prior and the current hemoglobin concentrations to remove cases for which hemoglobin decrease could have occurred for clinically significant reasons, such as acute blood loss.

All three criteria must be fulfilled to be predicted as a “potentially unnecessary” transfusion.

## 3 RESULTS

### 3.1 Model performance

The first performance evaluation on the simulated contamination in the training set using five‐fold cross‐validation is summarized in Supporting Information Figure 3. The ML pipelines were highly capable of distinguishing simulated contamination from real‐world results, demonstrating an area under the receiver operating characteristic curve (auROC) of 0.98 in the WashU set, and 0.99 in the Utah set, and an area under the precision‐recall curve (auPR) of 0.99 for both sets.

**FIGURE 2:**
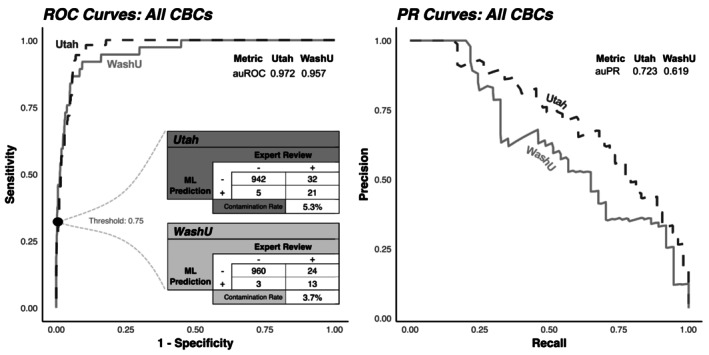
Performance evaluation in a set of 1000 expert‐labeled CBC trios. Receiver operating characteristic (ROC) curves and Precision‐Recall (PR) curves demonstrate the trade‐off in labeling performance across classification thresholds, with the area underneath them (auROC and auPR) summarizing total discriminatory potential. The inset element shows the confusion matrix for each set at a classification threshold of 0.75.

Next, real‐world performance was assessed using the validation sets from 1000 randomly selected CBC trios at each institution. The feature set with the best auPR was the approach that combined PCA with raw feature values (Supporting Information Figure 4), so only these models are described below. Compared to the expert review labels, models demonstrated point estimate auROCs of 0.972 and 0.957 in Utah and WashU sets, respectively, with auPRs of 0.723 and 0.619 (Figure 2). A binary classification threshold of 0.75 produced a sensitivity of 0.40, specificity of 0.995, positive predictive value (PPV) of 0.808, and negative predictive value (NPV) of 0.967 in the Utah set, and a sensitivity of 0.351, specificity of 0.997, PPV of 0.812, and NPV of 0.976 in the WashU set.

### 3.2 Model performance in the setting of RBC transfusion

**FIGURE 3:**
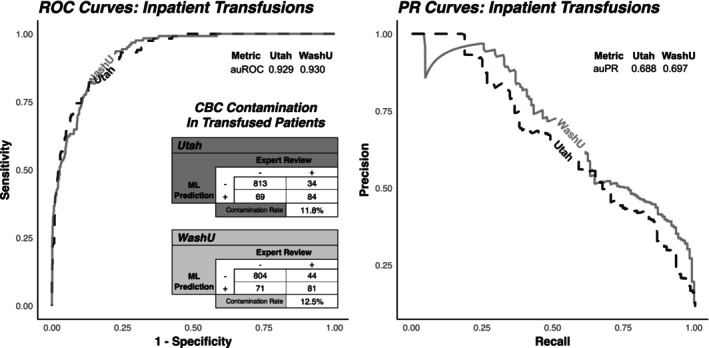
Performance evaluation in a set of 1000 expert‐labeled CBC trios with a RBC transfusion administered between the current and post‐CBC results. Receiver operating characteristic (ROC) curves and precision‐recall (PR) curves demonstrate the trade‐off in labeling performance across classification thresholds, with the area underneath them (auROC and auPR) summarizing total discriminatory potential. The inset element shows the confusion matrix for expert adjudication of contamination.

Given the importance of the post hemoglobin in model predictions, we sought to more comprehensively evaluate model performance on a common edge case: patients who received a RBC transfusion between their current and post specimens (Figure 3).

In CBCs that preceded a transfusion, the auROCs were 0.929 and 0.930, while the auPRs were 0.688 and 0.697 at Utah and WashU, respectively. Using the same specificity‐focused threshold of 0.75, the sensitivity was 0.712, specificity was 0.922, PPV was 0.549, and NPV was 0.96 in the Utah set. For the WashU set, the sensitivity was 0.648, specificity was 0.919, PPV was 0.533, and NPV was 0.948.

### 3.3 Rule performance for labeling a transfusion as potentially unnecessary

To evaluate the feasibility of applying the general criteria for labeling transfusions as potentially unnecessary, positive predictions were compared to expert chart review. 81 of 84 transfusions administered after positive predictions in the Utah set and 77 of 81 in the WashU set were deemed potentially unnecessary by manual review, for a combined PPV of 96%. Transfusions deemed justified despite predicted contamination included scenarios in which the post‐transfusion hemoglobin was still below 8 g/dL after the transfusion, there was active intraoperative or postoperative bleeding, and acute symptoms consistent with anemia were present.

### 3.4 Application to a full year of CBC results

The validated models were applied to the full, unlabeled dataset of all CBC trios collected in 2024 (Supporting Information Figures 5 and 6) at each institution. At Utah, of 288,204 CBCs performed, 110,299 (38%) had a prior and post result upon which a prediction could be made. At WashU, 322,205 (46%) of 691,118 CBCs were eligible for prediction.

**FIGURE 4:**
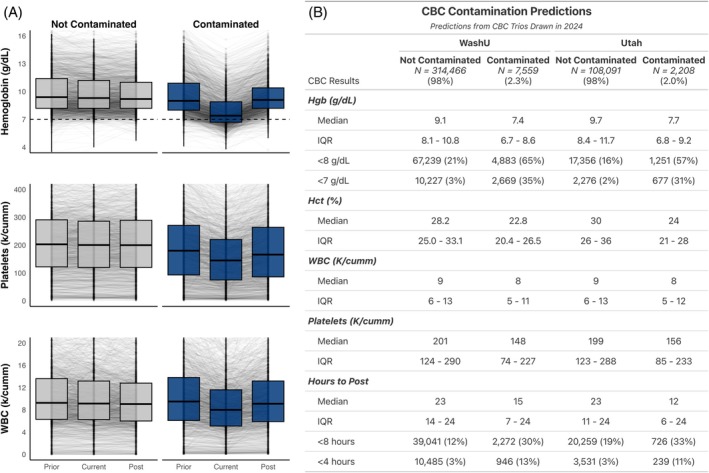
(A) Hemoglobin concentration, platelet count, and WBC count across the three time points (prior, current, post) for the validation set of CBC trios drawn in 2024. CBCs predicted as not contaminated are highlighted in gray (left), while those predicted as contaminated demonstrate the anomaly‐with‐resolution pattern and are highlighted in blue (right). Each boxplot represents the median and interquartile range, with lines extending across the middle 95th percentile. The dotted line across the hemoglobin plot indicates a hemoglobin concentration of 7 g/dL. (B) Hemoglobin, hematocrit, WBC count, and platelet count from the 2024 validation set are summarized in order to compare Contaminated and Not Contaminated specimens at each institution. “Hours to Post” represents the amount of time between the current and post samples, which is notably shorter for predicted contaminated samples at both institutions.

Of CBC trios that were reported into the electronic medical record in 2024, 2208 (2.0%) and 7559 (2.3%) were predicted as contaminated at Utah and WashU, respectively. A summary of these CBC trios is shown in Figure 4.

A total of 13,298 and 44,859 transfusions were extracted from the blood product administration modules at Utah and WashU, respectively, with 7632 (57%) and 27,980 (62%) having a matched CBC trio for a contamination prediction to be made. Of these, 686 (9%) and 1690 (6%) of transfusions for which a prediction could be made were flagged as potentially unnecessary using the retrospective rule set described and validated above.

### 3.5 Clinical features associated with unnecessary transfusion

**FIGURE 5:**
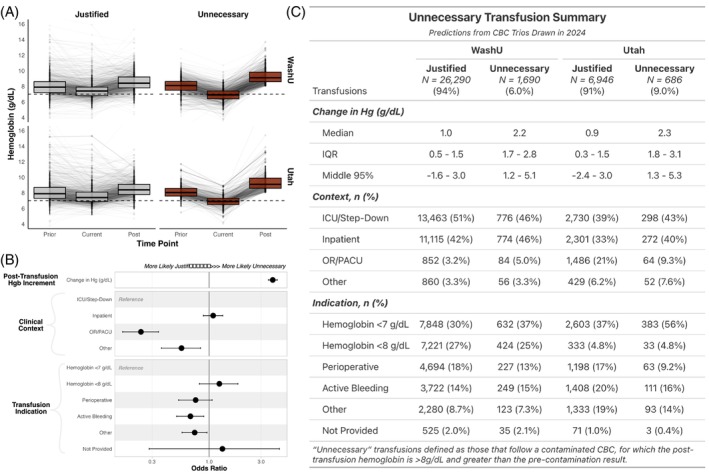
(A) Hemoglobin concentration across the three time points (prior, current, post) for the CBC trios drawn in 2024 with justified transfusions (gray, left) and those with potentially unnecessary transfusions (red, right) at each institution. Each boxplot represents the median and interquartile range, with lines extending across the middle 95th percentile. The dotted line indicates the most common hemoglobin threshold for RBC transfusion of 7 g/dL. (B) Odds ratios for factors contributing to the predicted necessity of transfusion (odds ratios above 1.00 suggest a higher likelihood that any given transfusion was labeled potentially unnecessary, as compared to the most frequent label for each feature (patients in intensive care units and transfusions with an indication of “hemoglobin <7 g/dL”). (C) Change in hemoglobin concentration from current to post (g/dL), clinical context, and indication for transfusion for those cases with justified vs. potentially unnecessary transfusions.

CBC trios predicted as contaminated demonstrated the expected anomaly‐with‐resolution pattern, where dilutional decreases are followed by commensurate increases across each analyte (Figure 5A). This pattern is accentuated in hemoglobin concentrations for the subset that received an RBC transfusion, which were more likely to drop below 7 g/dL in their current CBC than the non‐transfused and non‐contaminated counterparts.

The clinical contexts and clinician‐provided transfusion indications associated with potentially unnecessary transfusions are shown in Figure 5B, and summarized in Figure 5C. Larger post‐transfusion hemoglobin increments were more likely to be labeled potentially unnecessary, while transfusions administered peri‐ or intra‐operatively were significantly less likely to be labeled unnecessary compared to those given in the critical care or inpatient wards. Similarly, transfusions for which the given indication was “Active bleeding” or was provided as free text were also less likely to be labeled as potentially unnecessary.

### 3.6 Estimating contamination severity

**FIGURE 6:**
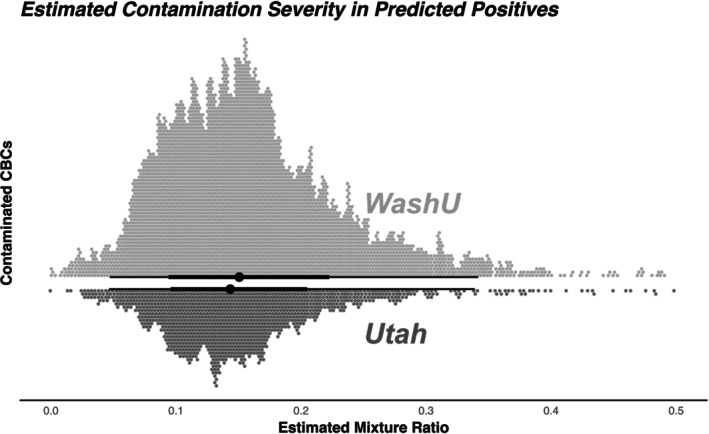
Estimated contamination severity in the 9767 contaminated real‐world CBC trios. Each point represents one predicted contaminated specimen, with those light gray points above the axis representing cases at WashU, and those below the axis at Utah. At Utah, the median mixture ratio was 0.13, with 1669 (76%) above 0.10, and 275 (13%) above 0.20. At WashU, the median mixture ratio was 0.14, with 4995 (66%) above 0.10, and 1046 (14%) above 0.20. The maximum estimated mixture ratio was 0.60 and 0.66 at Utah and WashU, respectively.

Contamination severity, as estimated by the mixture ratio from regression models trained on simulated contamination, was evaluated for all CBC trios predicted to be contaminated by the classification models described above. The median mixture ratio at Utah was 0.13, while the median mixture ratio at WashU was 0.14 (Figure 6). Notably, each of these trios was reported numerically—without an interpretive comment—into the electronic medical record as part of routine clinical care.

### 3.7 Orthogonal performance evaluation: Basic metabolic panels

**FIGURE 7:**
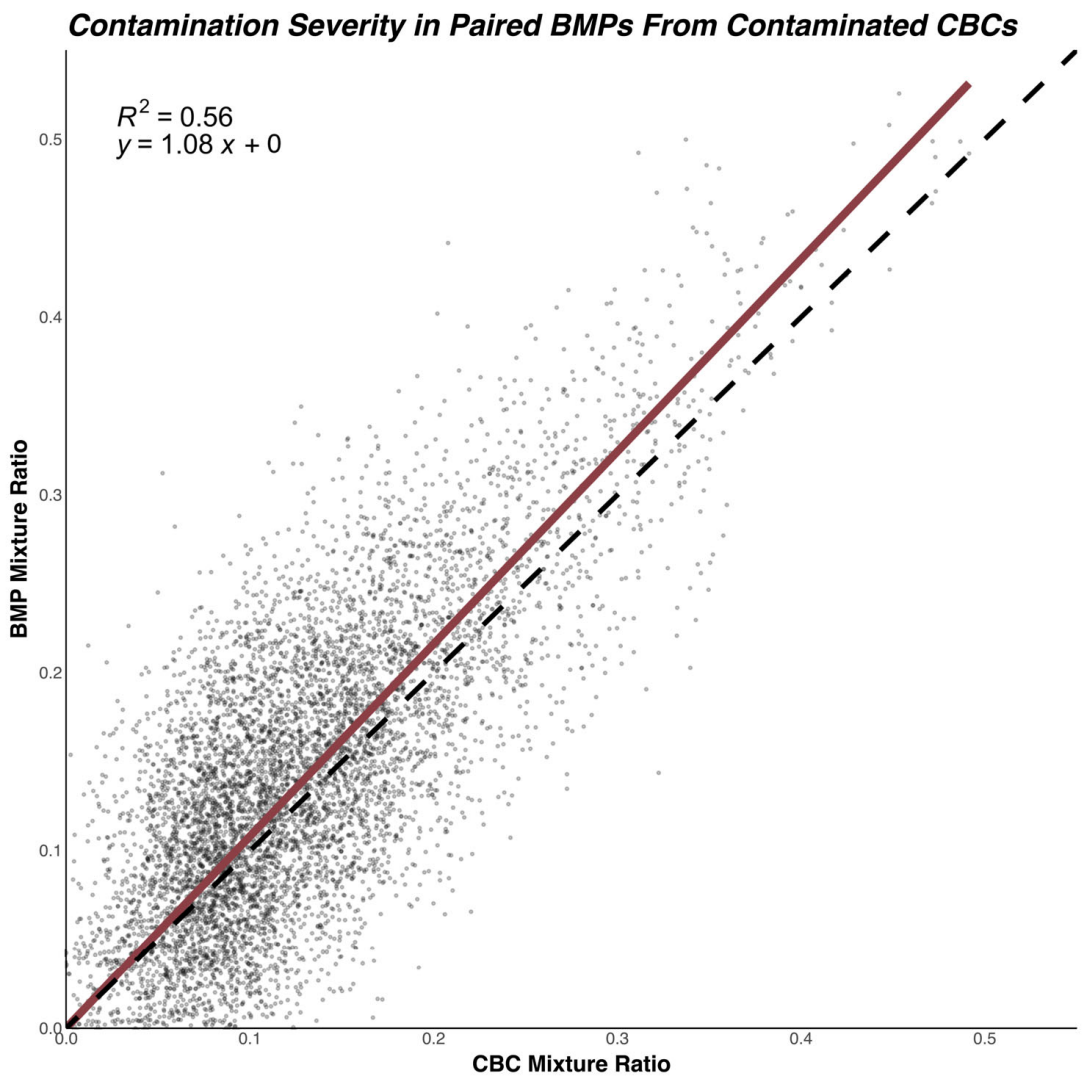
Mixture ratios in predicted contaminated CBCs compared to the mixture ratios of paired BMPs from WashU. CBCs predicted as contaminated with a concurrently contaminated BMP are shown in each point, with darker points corresponding to higher point density. Correlation between the mixture ratios for the CBCs and BMPs is quantified by the coefficient of determination (R^2^) and best‐fit line (solid red line), while the dashed line represent the identity line (y = x).

To evaluate these predictions with an orthogonal line of evidence, we hypothesized that if a CBC was predicted to be contaminated, and a BMP was drawn at the same time, it is likely that the BMP would also display evidence of contamination. If true, given that BMPs should be drawn prior to CBCs, these contamination events should be more pronounced in BMPs than in their paired CBCs. Estimated mixture ratios from paired CBCs and BMPs are shown in Figure 7.

Of the 4143 eligible pairs of CBCs demonstrating contamination with a mixture ratio of 0.10 or higher, 3410 BMPs (82%) also demonstrated evidence of contamination. In these contaminated pairs, a positive correlation was observed, with contamination severity in BMPs being slightly higher on average than their corresponding CBCs. The coefficient of determination was 0.56, while the line of best fit showed a slope of 1.08.

### 3.8 Explaining model and case predictions

**FIGURE 8:**
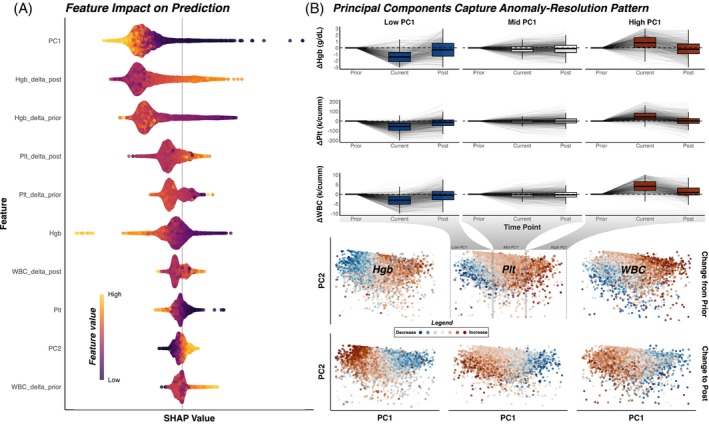
(A) A global evaluation of feature importance using Shapley Additive Explanation (SHAP) values, in which features are listed in decreasing order of median magnitude of their contribution. Each point represents one CBC trio in the test set. Points to the right of the center line suggest that value increased the model's predicted probability, while lighter points represent trios where that feature had a higher value. (B) The anomaly‐with‐resolution pattern as a function of principal component (PC) tertiles, top. Bottom, PC1 and 2 for each CBC trio, colored by change from prior and change to post for hemoglobin (Hgb), platelet count (Plt), and white blood cell count (WBC). Red points represent increases, while blue points represent decreases.

To better interrogate how the models generate their predictions, explainability metrics were calculated using SHAP values. SHAP values estimate each individual feature's contribution to the overall predicted probability in relation to all other feature values for each input sample. These can be aggregated across all predictions to provide a more “global” view of which features exert the strongest influence on predictions (Figure 8A) across the full, real‐world test set.

Globally, the most contributory feature was principal component 1 (*PC1*), or the transformed projection of the axis of greatest variation in the simulated training data set, followed by additive contributions from the change in hemoglobin from current to post (*Hgb_delta_post*), then the change in hemoglobin from current to prior (*Hgb_delta_prior*). Larger decreases from prior and larger increases to post were correlated with higher predicted probabilities of contamination. Similarly, lower PC1 values were correlated with higher contamination probabilities, as the prinicipal components appear to effectively capture the anomaly‐with‐resolution pattern typical of contamination, while higher values correspond to an opposite pattern across the analytes (Figure 8B).

## 4 DISCUSSION

In this work, we provide a proof‐of‐concept approach to retrospectively evaluate the prevalence of a common preanalytical error in CBCs through simulation‐informed, supervised ML. We demonstrate three key findings: (1) ML can effectively identify IV fluid contamination in CBC trios, even in the setting of RBC transfusion; (2) Contamination likely occurs in ~2% of inpatient CBC trios and is equally prevalent across institutions; and (3) these events may be motivating a substantial number of potentially unnecessary RBC transfusions. To our knowledge, this study is the first to evaluate the potential for ML to provide scalable, robust, and generalizable assessment of the frequency and clinical impact of preanalytical errors in CBC results.

We focus on transfusion decisions made in close proximity to contamination events as a retrospectively measurable proxy for clinical impact. While clinical decision‐making around transfusion is complex, we approximate a definition for “potentially unnecessary transfusions” as those that followed a contaminated CBC, for which the post‐transfusion hemoglobin was greater than 8 g/dL, and greater than the prior. This definition aligns well with adjudications of clinically justified transfusions from expert review,^18^ and applies to 2376 transfusions administered in 2024 across both institutions. These potentially unnecessary transfusions represent 6%–9% of all inpatient transfusions for which a CBC trio was available. While this denominator captures the population at risk for misguided transfusion decisions due to contamination, and allows for a more “apples‐to‐apples” comparison across institutions, it does not capture all transfusions administered, most notably those without a CBC trio available for prediction. While observed contamination and unnecessary transfusion rates exceeded *a priori* expectations, several factors add confidence to these results. First, similar prevalences across institutions, despite heterogeneous patient populations and transfusion practices, add generalizability to these findings. Second, the orthogonal evidence observed in the frequency and correlation between contaminated CBCs and their paired BMP counterparts, combined with the biologically plausible explanation (BMPs are drawn first, by the same collector, likely in the same manner), adds a layer of robustness to spurious noise or clinical factors that may be specific to CBCs.

Finally, there is notable congruence between expected and observed feature importances estimated by SHAP. Although the inclusion of PCA makes the final pipeline inherently more difficult to explain than if it were excluded, by highlighting the extent to which PC1 effectively captures the anomaly‐with‐resolution pattern, the resulting pipeline remains adequately explainable. Given the appreciable performance impact of including both the raw features and the principal components from the simulated training set, we believe the benefits outweigh their concurrent explainability penalty. Taken together, all these factors support our confidence in the models' predictions and the magnitude of the issue.

However, there is ample room for optimization and validation of this approach. Future directions should include real‐world, silent, prospective evaluations where positive predictions are confirmed via ancillary testing. For example, if a specimen was suspected of dextrose contamination, measuring an extremely elevated glucose concentration should provide effective confirmation. Accruing this real‐world, high‐fidelity set of ground‐truth labels would be crucial for adequate performance evaluation prior to live deployment, and could even be used as a supplement to the simulation‐based training labels. Additionally, the approach described here applies institution‐specific models. The trade‐off between performance and generalizability that a multi‐institutional approach could provide is worthy of future exploration.

This simulation‐based, wholly retrospective approach is the most notable limitation to the work described above. The lack of a gold‐standard definition of contamination forced two suboptimal labeling approaches: the use of synthetic data to train the models, and manual chart review to measure their performance. Prior work has demonstrated the fallible nature of human‐generated contamination predictions in chemistry panels.^10^ We hypothesize that the same is likely true in CBC results, given the alternative explanations for and mimickers of the anomaly‐with‐resolution pattern, such as fluid resuscitation and contraction. Additionally, this approach is unable to reliably capture transfusions that were administered outside of routine clinical workflows, such as unscanned or emergency release units, which we roughly estimated at ~10% of inpatient transfusions. Not only are these events missing from the statistical analysis, but they may have also impacted the model training and manual chart review upon which these results rely. Appropriately capturing these events, accounting for them in future training sets, and evaluating their real‐world impact through prospective validation studies should be prioritized prior to implementation.

This retrospective, ML‐driven approach could provide value to laboratories by evaluating the scope and impact of the issue at an institution, identifying high‐value target areas for quality improvement projects, and measuring the realized impact of these efforts. Such projects could span the total testing process by focusing on improving education regarding proper phlebotomy technique, implementing better rules‐based systems for detecting erroneous measurements, and developing appropriate safeguards against misguided treatment decisions. The ultimate goal should be implementation of models that can detect erroneous results in real time, eliminating the possibility of downstream error cascades and patient harm. However, this approach would rely only on features available at the time a “current” CBC is analyzed. Developing and validating these real‐time models without availability of post results is a high‐value future direction worth pursuing.

While several limitations and optimizations will need to be addressed prior to clinical implementation of any solution, retrospective or real‐time, we believe the work described here is a crucial first step toward achieving these goals. We hope that, through a combination of ML tools and educational outreach, the extent and impact of this issue can be better characterized, and that laboratories and clinical services can collaborate to find effective solutions toward improving the quality of the care we provide our patients.

## CONFLICT OF INTEREST STATEMENT

The authors declare no conflicts of interest.

## Supporting information


**Data S1.** Supporting Information.
Supporting Information Tables 1–2: Validating the Simulation with In Vitro Mixing Studies.

**Supporting Information Figure 1:** Validating the Simulation.

**Supporting Information Figure 2:** Distribution of Mixture Ratios for Simulated Training.

**Supporting Information Figure 3:** Performance Summary on Simulated Data in Cross‐Validation.

**Supporting Information Figure 4:** Feature Set Performance Evaluation.

**Supporting Information Figure 5:** CBC Results Summary.

**Supporting Information Table 6A:** CBC Contamination Predictions.

**Supporting Information Figure 6B:** Unnecessary Transfusion Summary.

## Data Availability

The data that support the findings of this study are openly available in Transfusions for Contaminated CBCs at https://github.com/nspies13/transfusions_from_contaminated_cbcs.

## References

[1] Strathmann FG , Baird GS , Hoffman NG . Simulations of delta check rule performance to detect specimen mislabeling using historical laboratory data. Clin Chim Acta [Internet]. 2011;412:1973–1977. Available from: https://www.sciencedirect.com/science/article/pii/S0009898111003846 21782806 10.1016/j.cca.2011.07.007

[2] Lippi G , Betsou F , Cadamuro J , Cornes M , Fleischhacker M , Fruekilde P , et al. Preanalytical challenges—time for solutions. Clin Chem Lab Med (CCLM) [Internet]. 2019;57:974–981. 10.1515/cclm-2018-1334/html 30710481

[3] Aziz Ali A , Khalid A , Moiz B . Performance evaluation of delta checks for error control in a hematology laboratory. Int J Lab Hematol [Internet]. 2021;43:e118–21. 10.1111/ijlh.13402 33222421

[4] Choucair I , Lee ES , Vera MA , Drongmebaro C , El‐Khoury JM , Durant TJS . Contamination of clinical blood samples with crystalloid solutions: an experimental approach to derive multianalyte delta checks. Clin Chim Acta [Internet]. 2023;538:22–28. Available from: https://linkinghub.elsevier.com/retrieve/pii/S0009898122013456 36309069 10.1016/j.cca.2022.10.011

[5] Spies NC , Farnsworth CW . Impact and frequency of IV fluid contamination on basic metabolic panel results using quality metrics. J Lab Med [Internet]. 2024;48:29–36. 10.1515/labmed-2023-0098/html

[6] Farrell C‐J , Makuni C , Keenan A , Maeder E , Davies G , Giannoutsos J . A machine learning model for the routine detection of “wrong blood in complete blood count tube” errors. Clin Chem [Internet]. 2023;69:1031–1037. Available from: https://academic.oup.com/clinchem/article/69/9/1031/7227182 37473426 10.1093/clinchem/hvad100

[7] Graham BV , Master SR , Obstfeld AE , Wilson RB . A multianalyte machine learning model to detect wrong blood in complete blood count tube errors in a pediatric setting. Clin Chem. 2025;71:418–427. 39797417 10.1093/clinchem/hvae210

[8] Baron JM , Mermel CH , Lewandrowski KB , Dighe AS . Detection of preanalytic laboratory testing errors using a statistically guided protocol. Am J Clin Pathol [Internet]. 2025;138:406–413. 10.1309/AJCPQIRIB3CT1EJV 22912358

[9] Rios Campillo C , de Sanz Pedro M , Iturzaeta JM , Qasem AL , Alcaide MJ , Fernandez‐Puntero B , et al. Design of an algorithm for the detection of intravenous fluid contamination in clinical laboratory samples. Clin Chem Lab Med. 2025;61:2002–2009. 10.1515/cclm-2023-0200 37270688

[10] Spies NC , Hubler Z , Roper SM , Omosule CL , Senter‐Zapata M , Roemmich BL , et al. GPT‐4 underperforms experts in detecting IV fluid contamination. J Appl Lab Med [Internet]. 2023;8:1092–1100. Available from: https://academic.oup.com/jalm/article/8/6/1092/7272601 37702018 10.1093/jalm/jfad058

[11] Spies NC , Hubler Z , Azimi V , Zhang R , Jackups R , Gronowski AM , et al. Automating the detection of IV fluid contamination using unsupervised machine learning. Clin Chem [Internet]. 2024;70:444–452. Available from: https://academic.oup.com/clinchem/article/70/2/444/7470143 38084963 10.1093/clinchem/hvad207

[12] Yang J , Wen S , McCudden CR , Tacker DH . Selection of single‐analyte delta check rules with logistic regression for detection of intravenous fluid contamination in a clinical chemistry laboratory. J Appl Lab Med [Internet]. 2024;9:1001–1013. Available from: https://academic.oup.com/jalm/article/9/5/1001/7705372 38959067 10.1093/jalm/jfae066

[13] Spies NC , Militello L , Farnsworth CW , El‐Khoury JM , Durant TJS , Zaydman MA . Prospective and external validation of an ensemble learning approach to sensitively detect intravenous fluid contamination in basic metabolic panels. Clin Chem [Internet]. 2025;71:296–306. Available from: https://academic.oup.com/clinchem/article/71/2/296/7901063 39545815 10.1093/clinchem/hvae168

[14] Newbigging A , Landry N , Brun M , Proctor D , Parker M , Zimmer C , et al. New solutions to old problems: a practical approach to identify samples with intravenous fluid contamination in clinical laboratories. Clin Biochem. 2024;127–128:110763. 10.1016/j.clinbiochem.2024.110763 38615787

[15] Kracalik I , Mowla S , Basavaraju SV , Sapiano MRP . Transfusion‐related adverse reactions: data from the National Healthcare Safety Network Hemovigilance Module ‐ United States, 2013‐2018. Transfusion. 2021;61:1424–1434. 33880771 10.1111/trf.16362

[16] Mowla SJ , Sapiano MRP , Jones JM , Berger JJ , Basavaraju SV . Supplemental findings of the 2019 National Blood Collection and utilization survey. Transfusion [Internet]. 2021;61:S11–35. 10.1111/trf.16606 34337759 PMC8441766

[17] Carson JL , Stanworth SJ , Guyatt G , Valentine S , Dennis J , Bakhtary S , et al. Red blood cell transfusion: 2023 AABB International Guidelines. JAMA [Internet]. 2023;330:1892. https://jamanetwork.com/journals/jama/fullarticle/2810754 37824153 10.1001/jama.2023.12914

[18] Maucione C , McLamb N , Zaydman MA , Pearson LN , Metcalf RA , Spies NC . Intravenous fluid contamination in complete blood counts may lead to unnecessary red blood cell transfusions. Transfusion. 2025;65:1996–98. 10.1111/trf.18370 40739781

[19] R Core Team . R: a language and environment for statistical computing [Internet]. Vienna, Austria: R Foundation for Statistical Computing; 2023. https://www.R-project.org/

[20] Kuhn M , Wickham H . Tidymodels: a collection of packages for modeling and machine learning using tidyverse principles. [Internet]. 2020. https://www.tidymodels.org

[21] Wickham H , Averick M , Bryan J , Chang W , McGowan L , François R , et al. Welcome to the Tidyverse. Joss [Internet]. 2019;4:1686. 10.21105/joss.01686

[22] Wickham H . ggplot2: elegant graphics for data analysis. 2nd ed. Cham: Springer International Publishing; 2016.

[23] Sjoberg DD , Whiting K , Curry M , Lavery JA , Larmarange J . Reproducible summary tables with the gtsummary package. R J [Internet]. 2021;13:570. https://journal.r-project.org/archive/2021/RJ-2021-053/index.html

[24] Landau WM . The targets R package: a dynamic make‐like function‐oriented pipeline toolkit for reproducibility and high‐performance computing. J Open Source Softw. 2021;6:2959. 10.21105/joss.02959

[25] Lundberg S , Lee S‐I . A unified approach to interpreting model predictions. arXiv. 2017. https://arxiv.org/abs/1705.07874

