# Supplementary material for: Identification of IV fluid contamination in complete blood counts and subsequent unnecessary red blood cell transfusions using artificial intelligence
Source: Transfusion. 2026 Jan 8;66(3):469–80. doi: 10.1111/trf.70072 (PMC12983124; doi:10.1111/trf.70072)
Supplement: Supplementary file 1 — Data S1. Supporting Information. Supporting Information Tables 1–2: Validating the Simulation with In Vitro Mixing Studies. [file TRF-66-469-s008.docx]

Identification of IV fluid contamination in complete blood counts and subsequent unnecessary red blood cell transfusions using artificial intelligence - Supplement

[**Supplementary Tables 1-2:** Validating the Simulation with In Vitro Mixing Studies **2**](#_h1hgoib1ldtx)

[**Supplementary Figure 1:** Validating the Simulation **4**](#_8gh54h3uieqx)

[**Supplementary Figure 2:** Distribution of Mixture Ratios for Simulated Training **5**](#_41078nw9ynm6)

[**Supplementary Figure 3:** Performance Summary on Simulated Data in Cross-Validation **6**](#_e8cxogwovn4v)

[**Supplementary Figure 4:** CBC Train and Test Results Summary **7**](#_3hbl831911fz)

[**Supplementary Figure 5:** Summary of Results in Test Sets **8**](#_c4hb334g0brd)

[**Supplementary Figure 6:** Explaining Model and Case Predictions **9**](#_tyg69s7mzz7e)

##

## **Supplementary Tables 1-2:** Validating the Simulation with *In Vitro* Mixing Studies

Blood was drawn using sterile technique into EDTA tubes and inverted immediately several times to mix. Blood was then aliquoted into specimen tubes with IV fluid (NS or D5-NS) at the mixture ratios of 0.1, 0.3, and 0.5. Total blood volume in each specimen tube, including the control (mixture ratio of 0), was 1 mL. CBC without differential was evaluated for each sample 15 minutes after sample preparation on a Sysmex XN-Series Automated Hematology System. To account for possible matrix effects, and to simulate a more real-life setting to account for time for specimen transport, each sample was run in duplicate at 45 minutes after sample preparation.

Identical experiments using normal saline (NS) and 5% dextrose in normal saline (D5-NS) were performed over two days, with the first experiment utilizing blood from Donor A and second utilizing blood from Donor B. Results are shown in the tables below (Supplementary Table 1 and Supplementary Table 2) as Experiment 1 and Experiment 2, respectively.

The results from Experiment 1 and 2, at both 15 minutes and 45 minutes, are shown in the line plots (Supplementary Figure 1). The measured values (Measured NS and Measured D5-NS, solid lines) were plotted alongside the expected value calculated using the mixture ratios (Expected, dashed line).

Supplementary Table 1: Experiment 1

| 15 minutes | | | | | | | |
| --- | --- | --- | --- | --- | --- | --- | --- |
|  |  | **NS – mixture ratios** | | | **D5-NS – mixture ratios** | | |
|  | **Control** | **0.1** | **0.3** | **0.5** | **0.1** | **0.3** | **0.5** |
| **WBC** | 5.04 | 4.41 | 3.54 | 2.56 | 4.58 | 3.57 | 2.48 |
| **RBC** | 5.38 | 4.62 | 3.65 | 2.62 | 4.72 | 3.64 | 2.56 |
| **HGB** | 13.9 | 11.9 | 9.5 | 6.8 | 12.3 | 9.4 | 6.6 |
| **HCT** | 42 | 36.2 | 28.7 | 20.6 | 36.9 | 28.7 | 20.2 |
| **MCV** | 78.1 | 78.4 | 78.6 | 78.6 | 78.2 | 78.8 | 78.9 |
| **PLT** | 252 | 227 | 173 | 127 | 226 | 172 | 116 |
| 45 minutes | | | | | | | |
| **WBC** | 5.16 | 4.33 | 3.49 | 2.61 | 4.62 | 3.59 | 2.45 |
| **RBC** | 5.25 | 4.66 | 3.65 | 2.63 | 4.72 | 3.66 | 2.59 |
| **HGB** | 13.4 | 12 | 9.5 | 6.9 | 12.3 | 9.5 | 6.6 |
| **HCT** | 40.6 | 36.5 | 28.6 | 20.6 | 36.8 | 28.9 | 21.2 |
| **MCV** | 77.3 | 78.3 | 78.4 | 78.3 | 78 | 79 | 81.9 |
| **PLT** | 255 | 231 | 175 | 131 | 221 | 179 | 117 |

Supplementary Table 2: Experiment 2

| 15 minutes | | | | | | | |
| --- | --- | --- | --- | --- | --- | --- | --- |
|  |  | **NS – mixture ratios** | | | **D5-NS – mixture ratios** | | |
|  | **Control** | **0.1** | **0.3** | **0.5** | **0.1** | **0.3** | **0.5** |
| **WBC** | 7.01 | 6.32 | 4.96 | 3.63 | 6.35 | 4.84 | 3.31 |
| **RBC** | 4.26 | 3.79 | 2.92 | 2.08 | 3.81 | 2.92 | 1.99 |
| **HGB** | 13.4 | 11.9 | 9.2 | 6.6 | 12 | 9.2 | 6.4 |
| **HCT** | 36.3 | 32.5 | 25.2 | 18.1 | 32.7 | 25.5 | 17.7 |
| **MCV** | 85.2 | 85.8 | 86.3 | 87 | 85.8 | 87.3 | 88.9 |
| **PLT** | 226 | 203 | 163 | 112 | 204 | 159 | 112 |
| 45 minutes | | | | | | | |
| **WBC** | 6.92 | 6.14 | 4.8 | 3.54 | 6.43 | 4.83 | 3.39 |
| **RBC** | 4.21 | 3.78 | 2.9 | 2.09 | 3.76 | 2.91 | 2 |
| **HGB** | 13.3 | 11.8 | 9.2 | 6.7 | 11.9 | 9.3 | 6.3 |
| **HCT** | 35.9 | 32.4 | 24.9 | 18 | 32.1 | 25.7 | 19.2 |
| **MCV** | 85.3 | 85.7 | 85.9 | 86.1 | 85.4 | 88.3 | 96 |
| **PLT** | 227 | 204 | 162 | 115 | 209 | 159 | 112 |

*NS,* normal saline*; D5-NS,* 5% dextrose in normal saline*; WBC*, white blood cells; *RBC*, red blood cells; *HGB*, hemoglobin; *HCT*, hematocrit; *MCV*, mean corpuscular volume; *PLT*, platelets.

Units: *WBC*, K/cumm; *RBC*, M/cumm; *HGB*, g/dL; *HCT*, %; *MCV*, fl; *PLT*, K/cumm

## **Supplementary Figure 1:** Validating the Simulation

*NS,* normal saline*; D5-NS,* 5% dextrose in normal saline*; WBC*, white blood cells; *RBC*, red blood cells; *HGB*, hemoglobin; *HCT*, hematocrit; *MCV*, mean corpuscular volume; *PLT*, platelets.

Units: *WBC*, K/cumm; *RBC*, M/cumm; *HGB*, g/dL; *HCT*, %; *MCV*, fl; *PLT*, K/cumm.

##

## **Supplementary Figure 2:** Distribution of Mixture Ratios for Simulated Training

## **Supplementary Figure 3:** Performance Summary on Simulated Data in Cross-Validation

## **Supplementary Figure 4**: Feature Set Performance Evaluation

Model performance was evaluated using three individual feature sets in order to assess contributions of PCA. These three feature sets included raw features alone, PCA alone, and combined features plus PCA. PCA was performed on all input and added as engineered features in the PCA alone and Features + PCA sets. Model performance was then assessed by comparing results to the validation sets, and the set with the best auPR, Features + PCA, was selected for use.

## **Supplementary Figure 5**: CBC Train and Test Results Summary

##

## **Supplementary Figure 6**: Summary of Results in Test Sets
