# Supplementary material for: Identification of IV fluid contamination in complete blood counts and subsequent unnecessary red blood cell transfusions using artificial intelligence
Source: Transfusion. 2026 Jan 8;66(3):469–80. doi: 10.1111/trf.70072 (PMC12983124; doi:10.1111/trf.70072)
Supplement: Supplementary file 6 — Supporting Information Figure 5: CBC Results Summary. [file TRF-66-469-s003.docx]

Supplementary Figure 5: ***CBC Results Summary***

|  | **Utah** | | | | **WashU** | | | |
| --- | --- | --- | --- | --- | --- | --- | --- | --- |
| **Characteristic** | **Train (+)** N = 140,010 | **Train (-)** N = 138,437 | **Test (+)** N = 1,515 | **Test (-)** N = 108,784 | **Train (+)** N = 475,814 | **Train (-)** N = 469,920 | **Test (+)** N = 7,636 | **Test (-)** N = 314,389 |
| **Hgb (g/dL)** |  |  |  |  |  |  |  |  |
| *Median* | 7.0 | 9.5 | 7.7 | 9.7 | 6.7 | 9.0 | 7.4 | 9.1 |
| *IQR* | [5.8, 8.5] | [8.2, 11.6] | [6.8, 9.2] | [8.3, 11.7] | [5.6, 7.9] | [8.0, 10.6] | [6.7, 8.6] | [8.1, 10.8] |
| *<8 g/dL* | 94,109 (67%) | 26,681 (19%) | 833 (55%) | 17,774 (16%) | 357,382 (75%) | 106,676 (23%) | 4,876 (64%) | 67,246 (21%) |
| *<7 g/dL* | 69,371 (50%) | 4,121 (3%) | 458 (30%) | 2,495 (2%) | 268,665 (56%) | 18,860 (4%) | 2,625 (34%) | 10,271 (3%) |
| **Platelets (K/cumm)** |  |  |  |  |  |  |  |  |
| *Median* | 140 | 199 | 146 | 199 | 143 | 202 | 147 | 201 |
| *IQR* | [83, 211] | [120, 289] | [74, 227] | [123, 287] | [84, 214] | [123, 294] | [75, 227] | [124, 290] |
| **WBC (K/cumm)** |  |  |  |  |  |  |  |  |
| *Median* | 6.7 | 9.4 | 9.0 | 9.4 | 6.4 | 8.9 | 7.6 | 9.0 |
| *IQR* | [4.4, 9.8] | [6.4, 13.3] | [4.6, 12.9] | [6.3, 13.3] | [4.2, 9.4] | [6.0, 12.9] | [4.8, 11.0] | [6.2, 12.9] |
| **ΔHgb (Prior)** |  |  |  |  |  |  |  |  |
| *Median* | -2.70 | -0.20 | -1.60 | -0.10 | -2.50 | -0.10 | -1.50 | -0.10 |
| *IQR* | [-3.90, -1.80] | [-0.70, 0.30] | [-2.20, -1.10] | [-0.60, 0.30] | [-3.60, -1.70] | [-0.60, 0.30] | [-2.10, -1.00] | [-0.50, 0.30] |
| **ΔPlt (Prior)** |  |  |  |  |  |  |  |  |
| *Median* | -49 | -1 | -30 | -1 | -48 | 0 | -27 | 0 |
| *IQR* | [-87, -22] | [-16, 15] | [-61, -10] | [-17, 15] | [-87, -21] | [-15, 15] | [-55, -8] | [-14, 15] |
| **ΔWBC (Prior)** |  |  |  |  |  |  |  |  |
| *Median* | -2.5 | -0.1 | -1.5 | -0.2 | -2.30 | -0.10 | -1.30 | -0.10 |
| *IQR* | [-4.6, -1.0] | [-1.4, 0.9] | [-3.4, -0.1] | [-1.4, 0.9] | [-4.30, -0.90] | [-1.20, 0.80] | [-3.00, -0.10] | [-1.20, 0.90] |
| **ΔHgb (Post)** |  |  |  |  |  |  |  |  |
| *Median* | 2.40 | -0.10 | 1.40 | -0.10 | 2.30 | -0.10 | 1.30 | -0.10 |
| *IQR* | [1.60, 3.50] | [-0.50, 0.30] | [1.00, 2.00] | [-0.50, 0.30] | [1.50, 3.40] | [-0.50, 0.40] | [0.90, 1.90] | [-0.50, 0.30] |
| **ΔPlt (Post)** |  |  |  |  |  |  |  |  |
| *Median* | 50 | 1 | 27 | 1 | 50 | 1 | 23 | 1 |
| *IQR* | [23, 89] | [-12, 18] | [10, 51] | [-13, 17] | [22, 90] | [-12, 17] | [6, 48] | [-13, 16] |
| **ΔWBC (Post)** |  |  |  |  |  |  |  |  |
| *Median* | 2.04 | -0.17 | 1.11 | -0.18 | 2.00 | -0.10 | 0.90 | -0.10 |
| *IQR* | [0.70, 3.91] | [-1.35, 0.78] | [0.09, 2.79] | [-1.36, 0.76] | [0.80, 3.80] | [-1.20, 0.80] | [0.00, 2.30] | [-1.20, 0.80] |
