# Supplementary material for: Identification of IV fluid contamination in complete blood counts and subsequent unnecessary red blood cell transfusions using artificial intelligence
Source: Transfusion. 2026 Jan 8;66(3):469–80. doi: 10.1111/trf.70072 (PMC12983124; doi:10.1111/trf.70072)
Supplement: Supplementary file 7 — Supporting Information Table 6A: CBC Contamination Predictions. [file TRF-66-469-s001.docx]

Supplementary Table 6A: **CBC Contamination Predictions**

*Predictions from CBC Trios Drawn in 2024*

|  | **WashU** | | **Utah** | |
| --- | --- | --- | --- | --- |
| CBC Results | **Not Contaminated***N = 314,389*(98%) | **Contaminated***N = 7,636*(2.4%) | **Not Contaminated***N = 108,784*(99%) | **Contaminated***N = 1,515*(1.4%) |
| ***Hgb (g/dL)*** |  |  |  |  |
| Median | 9.1 | 7.4 | 9.7 | 7.7 |
| IQR | 8.1 - 10.8 | 6.7 - 8.6 | 8.3 - 11.7 | 6.8 - 9.2 |
| <8 g/dL | 67,246 (21%) | 4,876 (64%) | 17,774 (16%) | 833 (55%) |
| <7 g/dL | 10,271 (3%) | 2,625 (34%) | 2,495 (2%) | 458 (30%) |
| ***Hct (%)*** |  |  |  |  |
| Median | 28.2 | 22.9 | 30 | 24 |
| IQR | 25.0 - 33.1 | 20.5 - 26.6 | 26 - 36 | 21 - 28 |
| ***WBC (K/cumm)*** |  |  |  |  |
| Median | 9 | 8 | 9 | 9 |
| IQR | 6 - 13 | 5 - 11 | 6 - 13 | 5 - 13 |
| ***Platelets (K/cumm)*** |  |  |  |  |
| Median | 201 | 147 | 199 | 146 |
| IQR | 124 - 290 | 75 - 227 | 123 - 287 | 74 - 227 |
| ***Hours to Post*** |  |  |  |  |
| Median | 23 | 15 | 23 | 12 |
| IQR | 14 - 24 | 7 - 24 | 11 - 24 | 6 - 24 |
| <8 hours | 39,088 (12%) | 2,225 (29%) | 20,458 (19%) | 527 (35%) |
| <4 hours | 10,504 (3%) | 927 (12%) | 3,583 (3%) | 187 (12%) |
