# Supplementary material for: Identification of IV fluid contamination in complete blood counts and subsequent unnecessary red blood cell transfusions using artificial intelligence
Source: Transfusion. 2026 Jan 8;66(3):469–80. doi: 10.1111/trf.70072 (PMC12983124; doi:10.1111/trf.70072)
Supplement: Supplementary file 8 — Supporting Information Figure 6B: Unnecessary Transfusion Summary. [file TRF-66-469-s002.docx]

Supplementary Figure 6B: **Unnecessary Transfusion Summary**

*Predictions from CBC Trios Drawn in 2024*

|  | **WashU** | | **Utah** | |
| --- | --- | --- | --- | --- |
| Transfusions | **Justified**  *N = 26,272*(94%) | **Unnecessary**  *N = 1,708*(6.1%) | **Justified**  *N = 7,225*(95%) | **Unnecessary**  *N = 407*(5.3%) |
| ***Change in Hg (g/dL)*** |  |  |  |  |
| Median | 1.0 | 2.2 | 0.9 | 2.3 |
| IQR | 0.5 - 1.5 | 1.7 - 2.8 | 0.3 - 1.5 | 1.8 - 3.1 |
| Middle 95% | -1.6 - 3.1 | 1.2 - 5.1 | -2.3 - 3.3 | 1.2 - 6.3 |
| ***Context, n (%)*** |  |  |  |  |
| ICU/Step-Down | 13,460 (51%) | 779 (46%) | 2,851 (39%) | 177 (43%) |
| Inpatient | 11,104 (42%) | 785 (46%) | 2,428 (34%) | 145 (36%) |
| OR/PACU | 848 (3.2%) | 88 (5.2%) | 1,501 (21%) | 49 (12%) |
| Other | 860 (3.3%) | 56 (3.3%) | 445 (6.2%) | 36 (8.8%) |
| ***Indication, n (%)*** |  |  |  |  |
| Hemoglobin <7 g/dL | 7,846 (30%) | 634 (37%) | 2,762 (38%) | 224 (55%) |
| Hemoglobin <8 g/dL | 7,198 (27%) | 447 (26%) | 349 (4.8%) | 17 (4.2%) |
| Perioperative | 4,703 (18%) | 218 (13%) | 1,218 (17%) | 43 (11%) |
| Active Bleeding | 3,714 (14%) | 257 (15%) | 1,452 (20%) | 67 (16%) |
| Other | 2,286 (8.7%) | 117 (6.9%) | 1,372 (19%) | 54 (13%) |
| Not Provided | 525 (2.0%) | 35 (2.0%) | 72 (1.0%) | 2 (0.5%) |
| *"Unnecessary" transfusions defined as those that follow a contaminated CBC, for which the post-transfusion hemoglobin is >8g/dL and greater than the pre-contamination result.* | | | | |
